# EL-SLE: Efficient Learning Based Stride-Length Estimation Using a Smartphone

**DOI:** 10.3390/s22186864

**Published:** 2022-09-10

**Authors:** Mingcong Shu, Guoliang Chen, Zhenghua Zhang

**Affiliations:** School of Environment Science and Spatial Informatics, China University of Mining and Technology, Xuzhou 21116, China

**Keywords:** indoor positioning, stride-length estimation, CNN, LSTM, adaptive learning, smartphone sensors

## Abstract

The pedestrian stride-length estimation is a crucial piece of personal behavior data for many smartphone applications, such as health monitoring and indoor location. The performance of the present stride-length algorithms is suitable for simple gaits and single scenes, but when applied to sophisticated gaits or heterogeneous devices, their inaccuracy varies dramatically. This paper proposes an efficient learning-based stride-length estimation model using a smartphone to obtain the correct stride length. The model uses adaptive learning to extract different elements for changing and recognition tasks, including Long Short-Term Memory (LSTM) and Convolutional Neural Network (CNN) modules. The direct fusion method maps the eigenvectors to the appropriate stride length after combining the features from the learning modules. We presented an online learning module to update the model to increase the SLE model’s generalization. Extensive experiments are conducted with heterogeneous devices or users, various gaits, and switched scenarios. The results confirm that the proposed method outperforms other state-of-the-art methods and achieves an average 4.26% estimation error rate in various environments.

## 1. Introduction

Reliable stride-length estimation is essential for many applications, including health monitoring and Internet of Things (IoT) services [1,2,3]. Moreover, accurate Stride-Length Estimation (SLE) plays a necessary procedure in the Pedestrian Dead Reckoning (PDR) mechanism in indoor positioning research [4,5]. Significantly, PDR using smartphones’ Micro-Electro-Mechanical System (MEMS) is more flexible than the dedicated device, such as shoes’ and shank’s IMU. Regarding the PDR mechanism with a smartphone, stride length calculates the location directly, so the results determine the accuracy of the positioning system [6]. However, dynamic environments, motion states, and posture significantly express different inertial information. The low-cost MEMS are inherent with noticeable random noise, making it challenging to obtain robust stride-length estimation with off-the-shelf smartphones.

Many studies about pedestrian stride-length estimation are available in the literature. They show good performance with some assumptions, such as single scenario and average speed, resulting in the low generalization of the model in real life. Some studies have proposed an SLE model based on the users’ leg length, gender, or weight information. However, those methods manually set personalized parameters from the empirical formula [7,8,9].

To address the issue of changing states and devices or user heterogeneity, we proposed efficient learning-based stride-length estimation (EL-SLE) by using adaptive learning and online learning. The deep neural network can autonomously map the measurements to predicated results, and the task-orientation deep neural network (DNN) design is vital to better mapping functions. Inspired by the network for camera pose estimation [10], we proposed adaptive learning to extract multiple features for recognition tasks and temporal changing awareness tasks by using the CNN network and LSTM network, respectively. Furthermore, the online learning module updates the parameters of the CNN framework to improve the generalization of the SLE model in the new scenes. To the best of our knowledge, we are the first to propose an SLE model based on both CNN and LSTM to extract multiple features with an adaptive learning framework. Moreover, visual-based localization is adopted to obtain the ground truth for training data labeling without additional hardware. Our key contributions are as follows:

- We cast the stride-length estimation problem as an adaptive learning problem with multiple feature representations using CNN and LSTM modules. We leverage data preprocessing on the IMU measurements with autoencoders and preintegration to eliminate white noise and improve the efficiency of the network.
- We propose a convenient training data obtaining method based on visual-inertial odometry, determining accurate labels of IMU training data for the SLE model using a smartphone’s built-in camera.
- We proposed an online learning module that recognizes the current motion characters to update the network, automatically adjusting the mapping function for the user’s stride-length model using the aid of visual-inertial odometry.
- We conduct extensive experiments with various scenarios, motion modes, gaits, and devices/users, and the results show that the EL-SLE model outperforms the state-of-the-art methods.

The paper is organized as follows: Section 2 presents an overview of related work; Section 3 describes the proposed efficient learning-based stride-length estimation (EL-SLE) model by using adaptive learning and an online learning module; Section 4 verifies the performance of the proposed model with elaborate experiments; and Section 5 presents the conclusions and future work discussion.

## 2. Related Work

Accurate stride-length estimation is essential for field applications such as human motion monitoring, gait analysis for the elderly, and IoT service [11,12]. At the same time, various solutions, such as Vicon using the camera, depth-sensing camera, pressure sensor, etc., can provide the stride length [13,14,15,16]. However, the inertial sensor seems more suitable for SLE in our daily lives due to its low implementation cost and unlimited range of motion. This section briefly overviews some related works about inertial sensor-based stride-length estimation.

Inertial sensors are ubiquitous in mobile devices, and they can derive kinstate based on Newton’s second low or empirical formula. The SLE using inertial sensors is usually divided into two different approaches [17]: direct approaches based on double integration and indirect approaches based on a symmetrical gait model. The double acceleration integration is used to derive the step length using the traditional motion mechanism, and the one is to find how to establish the relationship between the statistical data with gait models and stride length. Regarding model theory and convenience, the direct approach may have more advantages than the indirect approach due to requiring no training data. However, noise and bias are inherent with the low-cost inertial sensors, resulting in cumulated error during the integration process. Therefore, the motion character of pedestrian walking is analyzed using heuristics to constrain the cyclical motion. Zero Velocity Update (ZUPT) has been proposed to reduce the error accumulation by resetting the motion vector when the foot is touching the ground during pedestrian walking [18,19,20]. ZUPT is employed with the foot-mounted sensor for better application, and the effect for the other body parts (such as pocket or handheld) is not significant during complex movement. Therefore, the direct-based stride-length estimation in the mobile device is not reliable in obtaining an accurate calculation using the integration methods.

According to the motion statistics and assumption for the stride-length estimation, the indirect approaches can be classified as empirical relationships [7,8], inverted pendulum [21,22], and statistical regression method, including acceleration-based methods [23,24], step frequency-based methods [25,26,27], angle-based methods [28,29] and multiparameter methods [30,31]. The training data collection and matching process are needed in these methods to learn the relationship between sensor information and the stride length. Moreover, the cyclic motion features are analyzed to obtain an accurate step length model [32] to remove the limitation of carrying the device for pedestrians. However, the extracted features tend to be different, and they cannot provide the generalization performance with the different pedestrians’ motions. Similarly, a context-based stride-length estimation is proposed to obtain motion features using a linear fusion of the stride frequency and acceleration variance [33]. The fatal stride-length error may come with the error of context recognition, and a context weighted model is proposed to estimate the stride length, and the different context probabilities are calculated as weight to compute the length [30]. Nonetheless, the manual classification based on prior information cannot cover all the pedestrian motion statement and scenarios.

Recently, regression-based and deep learning-based methods have been proposed to obtain accurate stride-length estimation [28,34,35,36]. The smartphone carrying modes are recognized to increase the robustness of the calculation [34]. For more training data and a reliable model, GPS information is used to label the data from the inertial sensors during pedestrian walking, and a hybrid multiscale CNN and recurrent neural network (RNN) are employed to regress the speed and time interval [35]. However, the model cannot be applied in indoor scenes due to lacking great GPS information for indoor data. According to sequential features, the denoising autoencoders and LSTM predicate the stride length [37]. Another solution for stride-length estimation is proposed by only using CNN [36]. However, these methods depend on the training data with pre-set walking characteristics which cannot apply to all gaits and environments.

Moreover, none have paid spatial and temporal features corresponding to recognition and modeling problems. The purpose of this paper is to employ a deep neural network to extract multiple features for the stride-length estimation, and the online learning module increases the robustness of the model by considering the heterogeneity of devices and users. Meanwhile, training data is required in the learning-based methods, and two methods are usually employed: segmentation with known total distance [9] and additional device aiding [37]. The first method is simple with accuracy, while it is not suitable for complex training in a large-scale environment. The other method requires an additional device, such as an optical motion capture system [38], a specialized Optojump system [39], and reliable foot-mounted sensors [40]. However, these methods require specific infrastructure and expensive devices. In this paper, the smartphone’s built-in camera obtains the ground truth using the visual-inertial odometry (VIO) solution [41,42], which is convenient for labeling the training data requirements.

**Figure 1:**
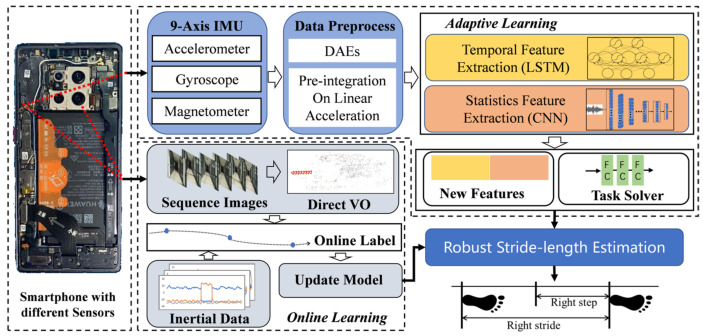
Flowchart of the efficient learning-based stride-length estimation.

We propose an efficient learning-based SLE model that combines adaptive learning and an online learning module to obtain a robust stride-length estimation with a smartphone. As shown in Figure 1, we take advantage of the neural networks to extract the statistic features and temporal features for multiple representations. Moreover, online learning maintains the model’s generalization for heterogeneous devices or users. In addition, we proposed a convenient method to obtain the training data for the SLE model using smartphones.

## 3. Algorithm Description

### 3.1. System Architecture

**Figure 2:**
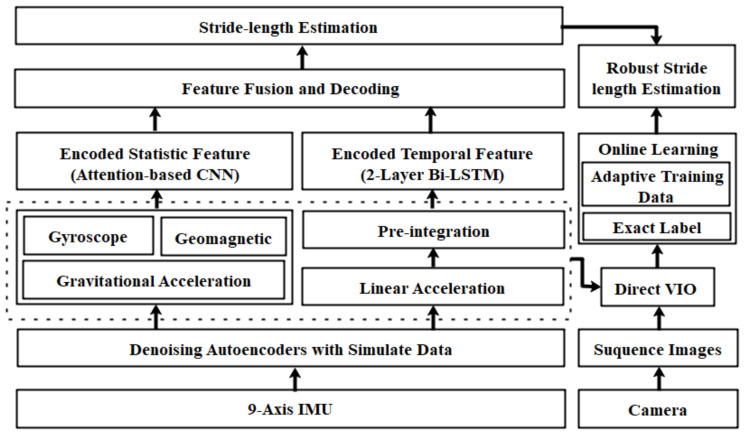
The system architecture of the efficient learning-based stride-length estimation model.

In this paper, we consider a robust stride-length estimation scenario where the pedestrian is carrying a smartphone, and their motion has no limitation in complex environments. The learning-based model maps the IMU measurements to the stride length for robust SLE, and the system architecture is shown in Figure 2. Firstly, we leverage denoising autoencoders to achieve data augmentation and employ preintegration to reduce computational complexity. Moreover, the adaptive learning module extracts the statistical and temporal features using CNN and LSTM, respectively. We employ a visual-based localization algorithm for the training data to provide reliable ground truth using only smartphones. Subsequently, we present the online learning module to update the model for improving the generalization of the SLE model in new scenarios.

### 3.2. Stride Length Estimation Based on Adaptive Learning

This section presents the theory and details of the adaptive learning framework for the stride-length estimation model. Our proposed learning framework consists of four parts, which are as follows: (1) data preprocessing that employs data augmentation and data preintegration to improve the efficiency of the network; (2) modified CNN is used to extract the statistical features representation for various scenarios; (3) a bidirectional LSTM network obtains features representation with temporal correlations and continuity constraints; and (4) the features fusion strategy, including a regression model and fully-connected layers, are presented to obtain the outputs of the model.

#### 3.2.1. Data Preprocessing with Denoising Autoencoders and Preintegration

**Figure 3:**
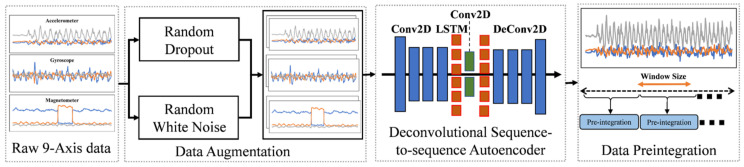
The framework of the data preprocessing.

To denoise the sensor readings from the low-cost IMU, the data-driven approach based on an autoencoder denoises motion signals, and we apply data augmentation for a better model with robust features. Furthermore, data preintegration is employed to reduce the time consumption of the feature extraction stage. As shown in Figure 3, the data preprocessing that we proposed consists of three steps: data augmentation, autoencoder, and preintegration. The specific process is shown in the following.

Considering the time series data from low-cost IMU, we use random dropout and random white noise to obtain the data augmentation [39,43]. The first method is that the part signal information is randomly removed and filled with zero in this part for the autoencoder module. The second method is adding random noise to the dropout part to extract the more significant features from the raw inertial data. The data augmentation can increase the model’s noise tolerance for signal reconstruction. Specifically, the accelerometer data and gyroscope data are added to the zero mean random normal noise, while we conduct the random dropout methods for the magnetometer data due to their noise being significantly related to environments [44]. One percent of the IMU data’s maximum value determines the noise’s standard deviation. Values are set as 0.16 and 20 for the accelerometer and gyroscope, respectively, as shown in the following:

(1) $${\hat{y}}_{i}^{x}=\left\{\begin{array}{c} y_{i}^{x}+N(u=0,\sigma =0.16),\;if\;x=Acc\\ y_{i}^{x}+N(u=0,\sigma =20),\;if\;x=Gyro \end{array}\right.$$

where $y_{i}^{x}$, ${\hat{y}}_{i}^{x}$ denote the input signal and output x by adding zero mean random noise.

The higher-level representations are extracted to obtain the target results and hidden underlying data-generation relationships to denoise the inertial data efficiently. Inspired by the related work [45] about motion signal reconstruction, we employ the deconvolutional sequence-to-sequence autoencoder to represent the IMU model accurately, improving the precision of pedestrian gesture recognition. As we know, specific information represents the IMU data, and it is challenging to leverage the handcrafted features to show the signal. Therefore, the data-driven approach learns the IMU data. As shown in the figure, in the variational autoencoder framework, we use 200 × 3 size samples from the IMU data with two kinds of augmentation forms as the input of the autoencoder, including an accelerometer, gyroscope, and magnetometer. For the structure of the neural network, we employ four Conv2D and four De-Conv2D layers with ReLu activation and 32 × 5 kernels. We use four stride sizes on the first and the last layers. The two stateless 64-unit LSTM layers are applied in the encoder and the decoder and dropout layers with a *p*-value of 0.5. Therefore, a stack of convolutional neural-network layers (Conv2D) extracts features, and a sequence of LSTM units perceives the temporal relationships. The autoencoder model takes the augmentation data h as input. Then, the encoder Enc(.) maps the data to representational features in the hidden layers, and the decoder Dec(.) maps the lower dimension space to generation data $\hat{h}$. The insight of an autoencoder is how to establish the optimal encode network that captures the samples’ dispersion characteristics and generates the data with the minimum error between input and output.

(2) $$\left\{\begin{array}{c} h=\left[\begin{array}{c} H(y_{i}^{x})\\ H({\hat{y}}_{i}^{x}) \end{array}\right],x=Acc/Gyro/Mag\\ \hat{h}=Dec(Enc(h)) \end{array}\right.$$

where h represents the hidden feature from IMU measurements. These higher-level features represent the IMU data due to its noise, and we employ the probabilistic generative model [45] to establish the relationship between features and the time-domain IMU samples in the following:

(3) $$\left\{\begin{array}{c} p_{\theta }(x,z)=p_{\theta }(x|z)p(z)\\ p(z)=N(0,Ι)\\ p_{\theta }(x|z)=N(\mu_{\theta }(z),\sigma_{\theta }^{2}(z)Ι) \end{array}\right.$$

where likelihood $p_{\theta }(x,z)$ quantifies the relationship between the observed IMU samples x and the hidden random variable z, and the prior p(z) quantifies the information about z before seeing samples. Mean $\mu_{\theta }(z)$ and variance $\sigma_{\theta }^{2}(z)$ represent the latent parameters in a neural network. This representation model uses the posterior $p_{\theta }(x|z)$ to infer z and obtain parameters that maximize the marginalized likelihood $p_{\theta }(x|z)$. As the encoder model in [45], the theory of variance inference approximates the posterior $q_{\phi }(z|x)$ with a similar and tractable distribution:

(4) $$q_{\phi }(x|z)=N(\mu_{\phi }(z),\sigma_{\phi }^{2}(z)Ι)$$

To train the parameters of the autoencoder model, we employ a weighted loss function that combines Kullback–Leibler divergence (KL) and Means Square Error (MSE) to train the model, and the representation is as follows:

(5) $$L=r\cdot (E_{MSE}[{\left(\hat{h}-h\right)}^{2}])+(1-r)\cdot E_{KL}[q(z|x)||p(z)]$$

where $E_{KL}$ represents the KL divergence describing the distribution similarity; $E_{MSE}$ measures the error of the input and output signal; and r is the weight values.

After denoising the autoencoder, the preintegration is adopted to improve the efficiency of the neural network, obtaining motion constraint variables by using IMU measurements in a pose graph. The 9D vectors constrain the orientations, velocities, and positions of keyframes based on the mathematical model of the IMU, and the measurements are used to propagate the object’s motion in the inertial frame using the recursive physic model in the following:

(6) Rn+1=RnExp((ω˜n−bg−ηg)^Δt)Vn+1=Vn+gΔt+Rn(a˜−nba−ηa)Δt

where $R_{n}$ and $V_{n}$ are orientation and velocity of the smartphone sensor in the world coordinate system, respectively, and Δt denotes the sampling time of the IMU. ${\tilde{\omega }}_{n}$ and $\tilde{a}{}_{n}$ represent the angular velocity and acceleration measurements from the gyroscope and accelerometer, which are with additive Gaussian noise $\eta_{g}$, $\eta_{a}$ and random walk bias terms $b_{g}$, $b_{a}$, respectively. Furthermore, the Exp(⋅) function in the above equation is the SO3 exponential map that converts the skew-symmetric members of the lie algebra so3 to their corresponding SO3 matrix, and (⋅)^ the operator converts a 3D vector into its skew-symmetric matrix representation.

The highly changing motion of the carrier may violate the constant world acceleration from the low-cost inertial sensor. Therefore, the consecutive IMU samples can be assumed to be constant in the body frame. Known as preintegration IMU factors, the consecutive IMU samples are compressed into a single vector using the constraint. Then, the state transition based on a preintegration constraint from the IMU measurements between time i and j can present as:

(7) $$\left\{\begin{array}{c} \Delta R_{ij}=\prod_{k=1}^{j-1}Exp(({\tilde{\omega }}_{k}-b_{k}^{g}-\eta_{k}^{g})\Delta t)\\ \Delta V_{ij}=\sum_{k=1}^{j-1}\Delta R_{ik}({\tilde{a}}_{k}-b_{k}^{a}-\eta_{k}^{a})\Delta t \end{array}\right.$$

where the initial state terms are moved to the left-hand side of the equation. In this paper, we only extract the pre-integrated features from the acceleration, which is the input in the LSTMs network, and the preintegration for the series signal can reduce the time consumption of the complex neural network without reducing signal quality.

#### 3.2.2. Recognition Feature Extraction with CNN Network

CNN is suitable for human activity recognition using hidden features from the inertial data in traditional works [45,46]. In this paper, the nine-axis data are used as input in the CNN to obtain the recognition features, and the inertial stride curve x with time interval T presents as:

(8) $$\begin{array}{l} x=(x_{1},x_{2},\cdot \cdot \cdot ,x_{t}),\\ x_{t}=(Acc_{t}^{x},Acc_{t}^{y},Acc_{t}^{z},Gyro_{t}^{x},Gyro_{t}^{y},Gyro_{t}^{z},Mag_{t}^{x},Mag_{t}^{y},Mag_{t}^{z}{)}^{T} \end{array}$$

where Acc, Gyro, Mag denote the output from preprocessing, including gravitational acceleration, gyroscope, and magnetometer.

**Figure 4:**
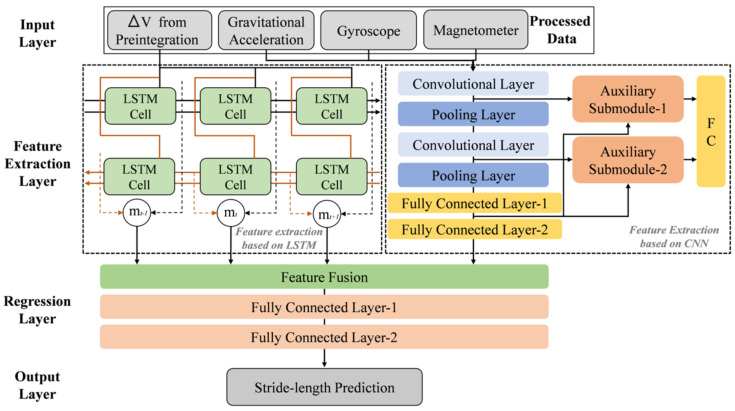
The efficient learning-based model architecture using spatial and temporal features representation for stride-length estimation.

Considering the noise of the low-cost IMU, we employ the modified CNN model [47] to amplify the prominent activity data and alleviate the impact of sensor noise. As shown in Figure 4, the modified CNN framework consists of two auxiliary submodules and an entire CNN pipeline which includes convolutional layers, pooling layers, and fully connected layers. The core of the submodule is the compatibility calculation between the local feature vector extracted in the middle layer of the CNN structure and the global feature vectors. The auxiliary submodule can provide the compatibility calculation for the feature extraction in the CNN pipeline, and the module’s detailed effect is how to integrate two vectors from the different features by using a compatibility function with a dot product. Then, a set of feature vectors $L^{s}=\left\{l_{1}^{s},l_{2}^{s},\cdot \cdot \cdot ,l_{n}^{s}\right\}$ is from a convolutional layer $s\in \left\{1,2,\cdot \cdot \cdot ,n\right\}$, and a global feature vector G connects with a set of feature vectors by using an additional operation. Then, a dot product is employed to represent the relationship between feature vectors and a weight vector u:

(9) $$c_{i}^{s}=\langle u,l_{i}^{s}+G\rangle ,i\in \left\{1,2,\cdot \cdot \cdot ,n\right\}$$

where $c_{i}^{s}$ represents the compatibility score. Then, the normalized form can be obtained by a Softmax function:

(10) $$\left\{\begin{array}{c} a_{i}^{s}=\frac{\exp(c_{i}^{i})}{\sum_{j}^{n}\exp(c_{i}^{s})}\\ A^{s}=\left\{a_{1}^{s},a_{2}^{s},\cdot \cdot \cdot ,a_{n}^{s}\right\} \end{array}\right.$$

Then, the normalized compatibility score $A^{s}$ provides a single vector $g^{s}$ for each layer s by using the element-wise average method:

(11) $$g^{s}=\sum_{i=1}^{n}a_{i}^{s}\cdot l_{i}^{s}$$

Subsequently, the calculation value $g^{s}$ replaces the global feature g, and the new feature vector presents as:

(12) $$g=\left[g_{1},g_{2},\cdot \cdot \cdot ,g_{n}\right]$$

In the auxiliary submodule of the CNN, the compatibility score $A^{s}$ represents the probability of the region where the pedestrian motion shows significant changes, and the auxiliary submodule represents the feature map. The weighting parameters in the module can enhance salient features and weaken insignificant features. CNN plays a significant role in extracting the statistical features for recognizing different gaits and scenes, and the experimental result in Section 4 has verified the performance.

#### 3.2.3. Temporal Features Extraction with LSTM Network

For the IMU data’s temporal features, the LSTM network is designed to handle time-series signals and capture the long-range dependencies in the sequential data. Unlike the direct LSTM network [40], we employ the bidirectional LSTM network to establish the relationship between the current statement and the front or back part statement.

As shown in Figure 4, the bidirectional LSTM network contains two layers, a forward layer and a backward layer, which are composed of the primary LSTM cell [48], and each cell process a sample by the forget gate, input gate, and output gate structure which are identical with the structure of works [49,50]. The input vector and long-term state represent as $x_{t}$ and $c_{t}$ at time-step t. $h_{t}$ and $h_{t}{}^{\prime }$ represent the recurrent hidden states of the forward and backward layers on the data sequence. For the hidden features provided by bidirectional layers, we employ the hidden states of the two layers to connect with concatenating setting $m_{t}$ at the time t, which is taken as the final recurrent hidden state of the network at a time t, as shown in the following:

(13) $$m_{t}=[h_{t},h_{t}{}^{\prime }]$$

Then, all recurrent hidden states are combined with the output of the LSTMs layers, which preserve the temporal features at all time steps, and the output matrix $O_{LSTM}$ can be written as:

(14) $$O_{LSTM}={\left[m_{1},m_{2},\cdot \cdot \cdot ,m_{n}\right]}^{T}$$

where n is the sequence length, and the max-pooling is employed to reduce redundant items for significant features.

#### 3.2.4. Regression Based on the Fusion Features

We now combine the two high-level feature representations by the CNN and LSTM network from the raw IMU data. The extracted temporal and spatial features generate more comprehensive and distinctive fusion features. The direct fusion approach is simpler and more efficient than the soft and hard fusion approaches [51]. Due to the features from the same sensor modality channels, we employ a straightforward approach to obtain the features fusion strategy using multi-layer perceptions (MLPs). The e regression layer provides the predicated stride length. As shown in Figure 4, once the hidden feature is determined, the nonlinear function has established a map from the feature vectors to the stride length using the training data. The error loss function L(D,G) is how to achieve the minimization for the regression layer between prediction and ground-truth of stride length, as expressed as:

(15) $$\left\{\begin{array}{c} \hat{y}=G(g_{direct}(a_{LSTM},a_{CNN}))\\ L(D,G)=\frac{1}{2N}\sum_{i=1}^{N}{\left(y_{i}-{\hat{y}}_{i}\right)}^{2} \end{array}\right.$$

where $\left[a_{LSTM},a_{CNN}\right]$ denote an MLP function that concatenates features from the adaptive learning module, which are used to represent the sequential and statistical features from the CNN and LSTM network, respectively. $y_{i}$ denotes the ground truth of the stride length from the input D, $\hat{y}$ denotes the estimation result from the regression layer, and $G(g_{direct}(a_{LSTM},a_{CNN}))$ represents the regression module for output determination. The loss function we use is standard in training the neural network.

### 3.3. Vision-Aided Training Data Collection

In this section, we present the data collection process for offline training and confirm the approach’s feasibility. There are many ways to obtain the ground truth for labeling data, such as manual segmentation, Vicon System, and dedicated track [38,43,52]. However, these approaches have complicated operations and need high-cost equipment support.

**Figure 5:**
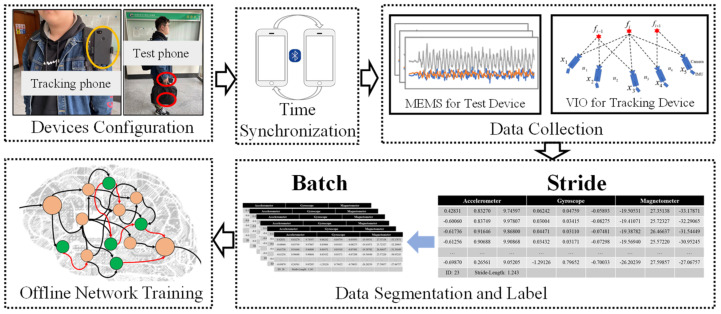
The framework of the vision−aided training data collection.

Recently, it has been possible to employ Visual-Inertial Odometry (VIO) with off-the-shelf smartphones [41,42], and this method can provide accurate locations once the loop detection is working. Inspired by the work [53], we propose a convenient approach to collect the training data using arbitrary smartphones. The framework of the collection approach is shown in Figure 5. We fix the smartphone to the chest with the camera facing out for motion tracking, and the other smartphone can be carried naturally, such as handheld gestures, being placed inside a pocket, or carried inside a bag, while the pedestrian conducts movement by normal walking, fast walking, running, or standing in various environments.

The proposed approach for training data collection consists of four parts, namely, device configuration, time synchronization, data collection, and data labeling. In the first step, the two smartphones are carefully calibrated, and we operate the process in each data sequence to guarantee the data quality. We correct the bias and scale errors using the method in [54]. Then, the tracking device is fixed to the chest with accessible equipment, as shown in the figure, and the test device is in the pre-set gestures. Secondly, an Android APP we developed is installed on the two smartphones for data collection, and the tracking device can connect with the test device through Bluetooth. Therefore, the system clocks of the device are with time synchronization. Thirdly, the two smartphones conduct the data collection. The IMU measurements are from the test devices, and the VIO algorithm provides an accurate position from the tracking device. We employ the sampling frequency of inertial sensors, 100 Hz, like the two smartphone settings in [40], and the 50 Hz for the camera frame rate. To generate the segmentation data for training, we employ a peak-based step detection algorithm [55] to count the steps during the motion and to determine the timestamp corresponding to the nodes of VIO. The steps are detected on the time *t* by using the algorithm. Then the nodes $p_{k}^{i}$ of the VIO can be obtained for the ground-truth $L_{g}^{K}$, as expressed in the following:

(16) $$\left\{\begin{array}{c} N=\left\{\left(t_{i},p_{1}^{i}),(t_{j},p_{2}^{j}),\cdot \cdot \cdot ,(t_{n},p_{N}^{n}\right)\right\}\\ p_{k}^{i}=(x_{K}^{i},y_{K}^{i},z_{K}^{i},q_{K}^{i})\\ L_{g}^{K}=\sqrt[{2}]{{\left(x_{K+1}-x_{K}\right)}^{2}+{\left(y_{K+1}-y_{K}\right)}^{2}} \end{array}\right.$$

where $p_{1}^{i}$ denotes the 6-DoF pose of node detected at *K* step of time $t_{i}$ in step detection algorithm.

Finally, we split the IMU data according to the timestamp of the above nodes. The training data is provided by the inertial data and stride length. To generate the fixed size and equal scale of input for the network, we set the 200 samples as the input size to cover various statements of pedestrian walking. For sequences longer than 200, we use the down-sampling strategy to process the original data and obtain the exact size of sequence samples. The ratio of downsampling is not an integer, while it cannot affect the learning process due to the neural network method with high-frequency samples input. They need to be filled with 0 to the samples when the sequences are shorter than 200. In each segmentation for training, the collection data $E_{K}$ contain 200 samples, nine channels for inertial sensors, and the corresponding ground-truth of stride length, as expressed in the following:

(17) $$\left\{\begin{array}{c} E_{K}=\left\{ID_{K},(e_{1},e_{2},\cdot \cdot \cdot ,e_{i},\cdot \cdot \cdot ,e_{200}),L_{g}^{K}\right\}\\ e_{i}=\left\{\left(Acc_{x},Acc_{y},Acc_{z}),(Gyro_{x},Gyro_{y},Gyro_{z}),(Mag_{x},Mag_{y},Mag_{z}\right)\right\} \end{array}\right.$$

where $ID_{K}$ denotes the identifier of $K^{\mathrm{th}}$ the segment, and $e_{i}$ represents a 9-dimensional vector from the inertial sensor.

**Figure 6:**
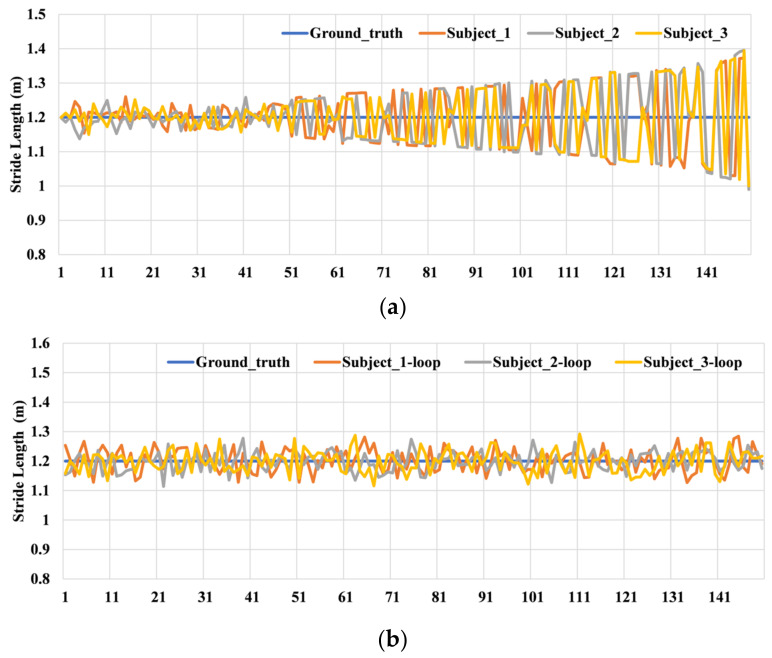
The stride-length estimation based on VIO without loop (**a**) and with loop detection (**b**).

After obtaining reliable locations from the convenient VIO-aided collection method, the labeled data are used as the input for network training processing. Figure 6 shows the stride-length estimation results of this approach when the pedestrian walks in the set path with or without loop detection, and the stride length is the same according to the floor tiles. From Figure 6, we find that the VIO can provide accurate length estimation once the loop is detected [42], and this approach needs walking in a loop for better ground-truth obtaining when we collect the training data. As shown in Figure 6, the errors of length estimation are almost less than 5 cm from the VIO with loop detection, which is enough to provide the ground truth for the model training.

### 3.4. Online Learning Module for Model Updating

**Figure 7:**
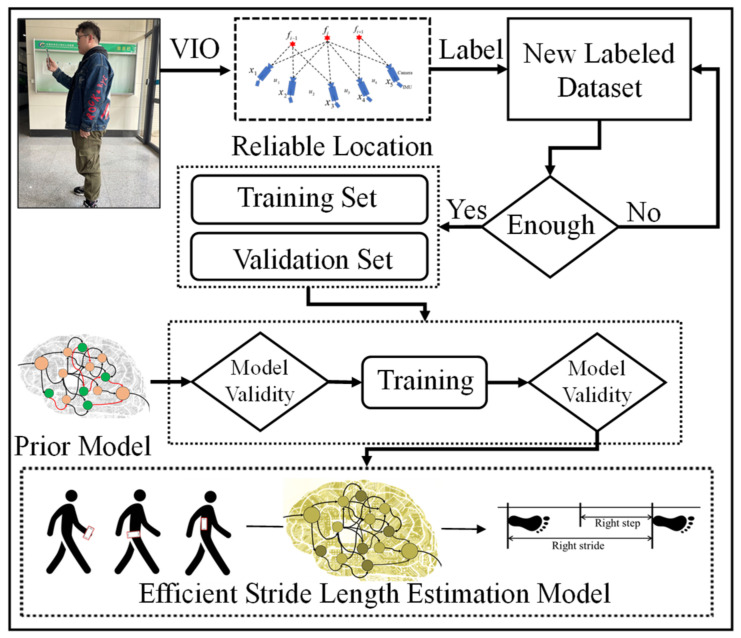
The framework of the updated SLE model based on online learning.

The online learning module is presented in this section to update the stride-length estimation model. Online learning can evolve the model to improve the robustness of neural networks [40,56]. It is essential to verify the offline SLE model and update the parameters of DNN for new data. We employ visual localization to obtain the new labeled data, and the model updating framework is performed to analyze the validity of the offline model. The framework of the online learning module is shown in Figure 7.

Moreover, we employ the VIO to the correct length of training data in this online learning module. The pre-correction mechanism obtains the ground truth. Specifically, the pedestrian holds a mobile phone with the camera open for a few seconds, as shown in the figure, and the visual and inertial data are from VIO and SLE, respectively. The results from the offline SLE model were compared with the step length from the visual-based method, and the model error $D_{\mathrm{mod}el}$ was obtained, as shown in the following:

(18) $$D_{\mathrm{mod}el}=\frac{1}{N}\cdot \sum_{i=1}^{N}\left(EL_{i}-L_{i}^{VIO}\right)$$

where N is the stride number, $EL_{i}$ and $L_{i}^{VIO}$ denote the predicted stride length and the ground-truth from VIO aiding of $i^{\mathrm{th}}$ stride.

When the difference is less than a particular threshold value, we believe the offline training model; otherwise, we use the new IMU data to update the model. The specific procedure for online learning presents in the following Algorithm 1:
**Algorithm 1:** The updating model procedure based on online learning1: **Input:** IMU data, VIO-based locations, and offline SLE model2: **Output:** a robust model for stride-length estimation3: //Online test data obtained from a smartphone4: IMU data segment according to the step detection algorithm, and remove the first segments5: **For** each test segment **do**6:  Calculating the ground truth of stride length from the statements based on VIO in Equation (16)7: **End for**8: Constructing the label data of each online segment as the form in Section 3.39: //Offline model verfication or updating10: **If** the size of the online test data is enough **then**11:  Evaluate the validity of the offline model according to Equation (18) by using online IMU data12:  **If** difference ≥ threshold (low reliability of offline model) **then**13:   Input the online test data as training data for the online learning framework and update the current SLE model14:   Obtain the new online test data when keep walking and evaluate the validity of the updating model according to Equation (18)15:   **if** difference < threshold (high reliability of offline model) **then**16:    Return the updating model from the online learning17:   **Else**18:    Return the offline SLE model after validity with new test data19:   **End if**20:  **End if**21: **End if**

In short, the online learning process can provide a pre-correction mechanism to reduce the offline model failure caused by personnel or equipment heterogeneity. At the same time, the online learning model is different from the offline model, which requires a large amount of training data. This online learning model only adjusts the CNN module parameters of the original model to adapt to the current users and output better step estimation results. Therefore, this process is a short learning process for learning new equipment or walking habits to improve the model’s generalization in new scenarios.

## 4. Experimentation and Evaluation

In this section, we first present the implementation details of the efficient learning-based stride-length estimation model. Then, the experimental setup, including equipment and environments, is described. We conduct various tests on the datasets, including different pedestrians, devices, gaits, and environments, to evaluate the proposed learning-based SLE method’s performance. The effectiveness of the proposed EL-SLE method is verified by comparing the localization results with the ground truth from the VIO-based localization. Last, we further apply our SLE model to the total distance and confirm the advantage of the proposed model.

### 4.1. Implementation

The efficient learning-based stride length estimation model was employed to provide reliable and robust pedestrian walking distance. Regarding the challenge of computation complexity, we employed client and server to calculate for model training and updating efficiently. A mobile application software designed in JAVA was implemented for Android smartphones, and the sampling rates of MEMS and vision were 100 Hz and 50 Hz. As depicted in previous research [38,53], the temporal history of 200 IMU samples segmentation for each inference. The window length of 200 can explain the movement changes during one step, balancing the performance and computational load. Keras [57] with pandas and Adam [58], a first-order gradient-based approach, are used for data management and algorithm optimization in this paper. We gathered abundant moving characteristics inside data. To avoid overfitting, we adopted the Dropout way [59], randomly dropping 25% units from the neural network to lower the risk during training, which improves the generalization ability.

### 4.2. Experimental Setup

**Figure 8:**
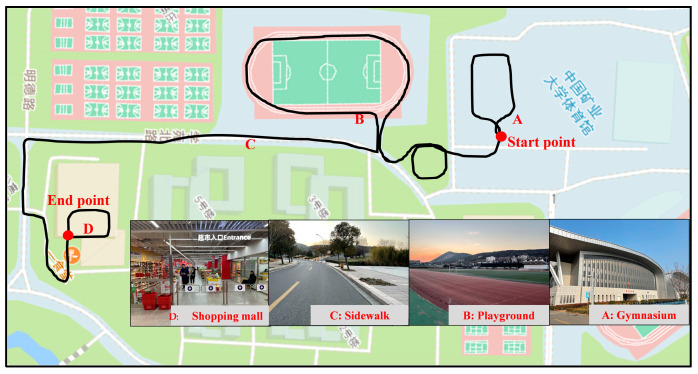
Pedestrian trajectory and test scenes description.

We conducted experiments in complex and changing scenes, including a gymnasium, sidewalk, playground, and underground shopping mall (the campus of the China University and Mining and Technology), which covers indoor and outdoor scenes, as shown in Figure 8.

**Figure 9:**
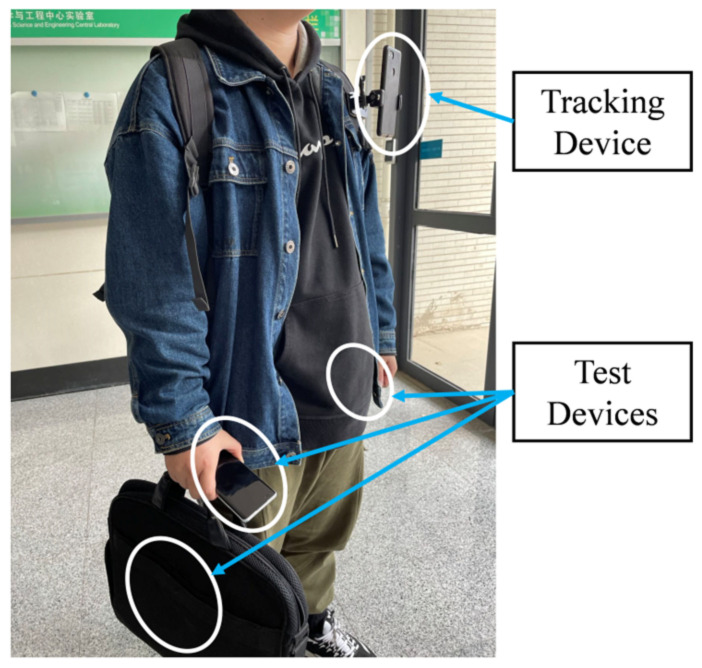
The scenario of data collection with different devices.

In the experiments, we used five Android smartphones, including four as test devices for IMU data collection and one as a tracking device for VIO; Figure 9 presents the scenario of the pedestrian collecting data. We utilized accurate visual-inertial odometry to obtain precise position information. For convenient comparison, we set Google Pixel XL 3 as the tracking device in the process, and the other four smartphones were set as the test devices. Five different users attached these devices to their body for data collection to reflect pedestrians moving in real life, and three gaits (slow walking, normal walking, and quick walking) were present during the process. Table 1 details the profiles of testers and smartphones. Our dataset has 20 sequences; we selected 15 sequences as training and the other 5 as tests. Table 2 presents the detail of sequences for the test. Our dataset’s total walking distance and recording process is over 31.5 km and 8.1 h, which can cover pedestrian movement distance in daily life.

### 4.3. Evaluation Metrics

We employ the stride-length error rate and walking-distance error rate to evaluate the proposed method. Furthermore, the localization errors are also used as an evaluation index according to the PDR mechanism. The stride-length error rate and walking-distance error rate are calculated by the following:

(19) $$\begin{array}{l} E_{s}=\frac{1}{N}\sum_{i=1}^{N}\left(\frac{\left|L_{e}^{i}-L_{g}^{i}\right|}{L_{g}^{i}}\cdot 100\%\right)\\ E_{cd}=\frac{\left|\sum_{i=1}^{M}L_{e}^{i}-\sum_{i=1}^{M}L_{g}^{i}\right|}{\sum_{i=1}^{M}L_{g}^{i}}\cdot 100\% \end{array}$$

where N represents the number of pedestrians walking, $L_{e}^{i}$ and $L_{e}^{i}$ represent the estimated length and the ground-truth of the $i^{\mathrm{th}}$ stride, respectively.

The following equation calculates the PDR-based localization error with different stride-length estimation models:

(20) $$\left\{\begin{array}{c} x_{i}=x_{i-1}+L_{e}^{i}\cos\theta_{e}^{i}\\ y_{i}=y_{i-1}+L_{e}^{i}\sin\theta_{e}^{i}\\ e^{i}=\sqrt{{\left(x_{i}-x_{VIO}\right)}^{2}+{\left(y_{i}-y_{VIO}\right)}^{2}} \end{array}\right.$$

where $\left(x_{i},y_{i}\right)$ denote the localization results from PDR. $L_{e}^{i}$ and $\theta_{e}^{i}$ denote the estimated stride length and heading estimation at $i^{\mathrm{th}}$ stride, respectively. $\left(x_{VIO},y_{VIO}\right)$ represent the precise position results from VIO.

### 4.4. Performance of Stride-Length Estimation Model

#### 4.4.1. Effect of Denoising Autoencoders and Preintegration

In this section, we analyze the effect of the data preprocessing, including data augmentation and preintegration, which is essential to obtain a reliable deep neural network for the SLE model. In the experiment, we compare the three stride-length estimation results: (1) the stride length results from the direct learning-based stride-length estimation model (marked as “L-SLE”); (2) the results from the learning-based stride-length estimation with data augmentation (marked as “L-SLE + DA”); and (3) the results from the learning based stride-length estimation with data augmentation and preintegration (marked as “L-SLE + DA + PI”). The three models are used to train the neural networks with the dataset, and the results are obtained from each neural network model. Table 3 illustrates the performance of the results from the three models. We find that the stride-length estimation results from the preprocessing, including “L-SLE + DA” and “L-SLE + DA + PI”, are better than the results from the direct learning manner, “L-SLE”. Compared with results from “L-SLE”, the mean error of stride length estimation results is reduced by 5% when using “L-SLE + DA” and “L-SLE + DA + PI”.

Meanwhile, the error rates from “L-SLE + DA” and “L-SLE + DA + PI” are 0.5% and 1.4%, and their results are less than that of “L-SLE”. Therefore, the accuracy of SLE can be improved when the raw IMU data is with augmentation or preintegration. In addition, it is evident that “L-SLE + DA + PI” is better than “L-SLE + DA” in both mean error and error rate, and the results show that the average mean error reduces from 5.8 cm to 5.7 cm, and the average error rate from 4.27% to 4.23%. Concurrently, compared with “L-SLE + DA”, “L-SLE + DA + PI” has less resource consumption to train for the neural network, reducing time consumption by about 0.15 ms/step because of the compact motion features vector by using preintegration processing.

#### 4.4.2. Effect of the Adaptive Learning

The effect of the adaptive learning we proposed is analyzed in this section. The other two single neural networks, LSTM and CNN, are used to compare with the proposed adaptive learning. The two networks based on LSTM and CNN are the same as the structure of the neural network proposed in Section 3.2. These compared methods depend on a single neural network as the framework in previous studies [50,60]. We use the dataset to train the three models, and five test sequences are used to test their performance. The performance of the different networks for stride-length estimation is shown in Table 4. We find that the LSTM + CNN have the best performance in both max error and error rate. Compared to the other single networks, LSTM and CNN, the average max stride-length estimation error decreased by 4% and 25%, respectively, and the proposed network can significantly decrease the average error rate of stride-length estimation by nearly 5% and 10%.

Meanwhile, the results from the LSTM-based SLE are better than that from CNN; the reason is due to the advantage of RNN, which can efficiently extract the temporal feature to remember the feature relationship with time. However, CNN is suitable for extracting the features for the recognition task. Considering the random changes in pedestrian walking modes and scenes, we proposed adaptive learning to extract multiple features with the CNN and LSTM modules.

**Figure 10:**
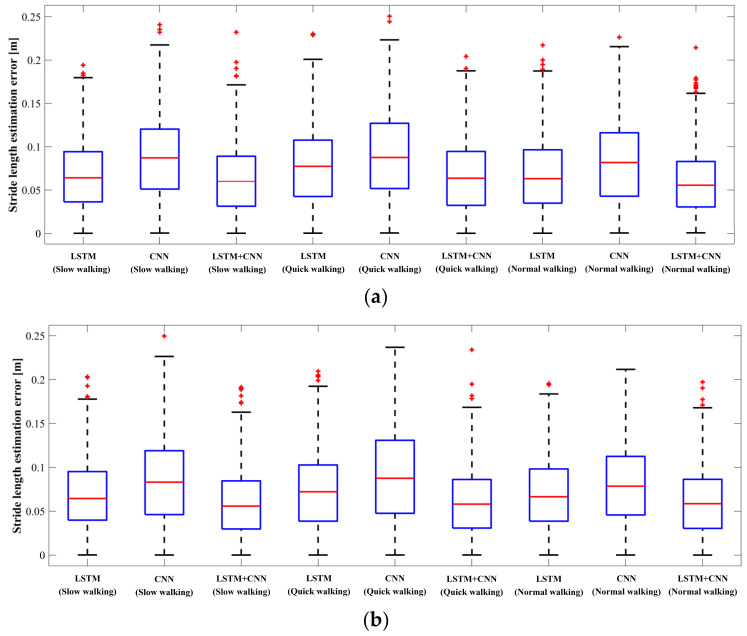
Box-plot of stride-length estimation error in three walking modes (slow walking, normal walking, and quick walking) with sequences #7 (**a**) and #11 (**b**).

**Figure 11:**
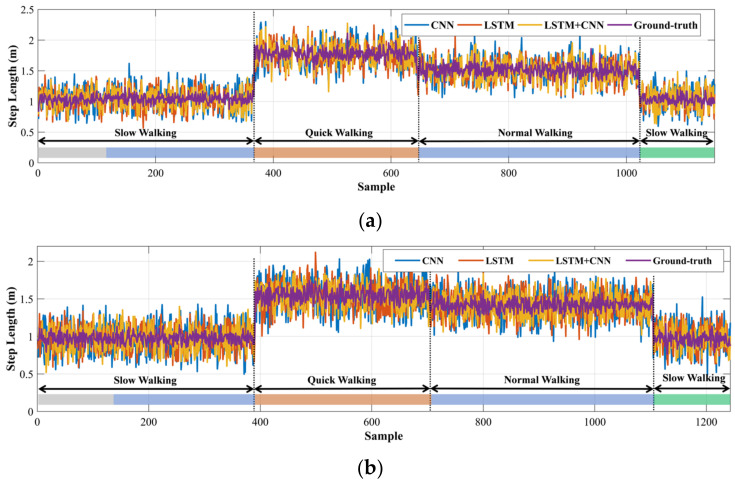
Qualitative comparison of the stride-length estimation results with a single network, CNN and LSTM, and a combined network, LSTM + CNN, on the sequence #7 (**a**) and #11 (**b**) in different scenes (indoor stadium, sidewalk, playground, and supermarket, are represented by color strip corresponding to gray, blue, orange, and green, respectively).

In the experiment, according to sequences #7 and #11, we analyze the stride length estimation results of LSTM, CNN, and LSTM + CNN in various walking modes and scenes. Figure 10 presents the box-plot of stride-length estimation error in various walking modes, including slow, normal, and quick walking. Furthermore, the qualitative results of three networks on sequences #7 and #11 are illustrated in Figure 11. It is clear that LSTM + CNN shows the best performance, and the CNN-based stride length method has the least accuracy, especially on sequence #11 with quick-walking mode. Meanwhile, LSTM + CNN is more stable than the other methods, and it can provide an excellent performance of stride-length estimation regardless of male and female users because of the multiple motion feature extraction. Based on the qualitative results in Figure 11, it is evident that the LSTM + CNN is close to the ground truth for sequences #7 and #11 in different scenes, while the CNN model’s estimations are relatively more jittery on sequence #7. Therefore, the proposed LSTM + CNN model can provide accurate and stable stride-length estimation in various scenes compared with single networks.

#### 4.4.3. Comparison between Normal and Updated Models

**Figure 12:**
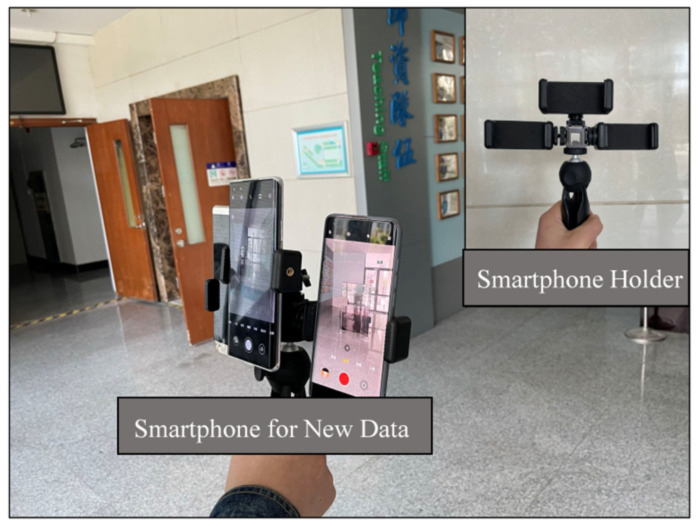
The new sequences collection using the holder.

In this section, we experiment to verify the effect of the online learning module in the proposed SLE model in Section 3.4. The new test sequences, sequences #21, #22, and #23, are collected from the new smartphones held by a new tester when walking on the same path, so we call these new IMU data unseen data, and the scenario of new data collection is shown in Figure 12. The unseen data we collect is used to test the generalization of the SLE model. For convenience, we kept smartphones facing forward to collect online labeled data for a few seconds. The process revises the SLE using the online learning module illustrated in Section 3.4. Table 5 summarizes the offline and updated models’ stride-length estimation results. The updated model has better performance than the offline model. Compared with the offline model, the mean error decreases from 6.8 cm to 5.9 cm on average, and the mean error rate decreases from 4.33% to 4.26%.

**Figure 13:**
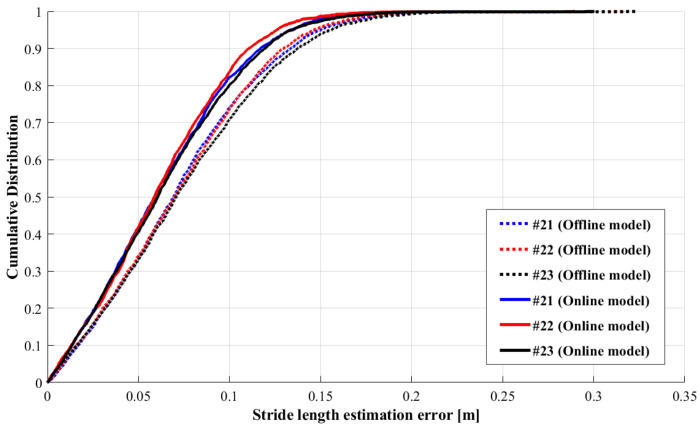
Qualitative comparison of stride-length estimation error between the offline model (SLE model without online learning module) and updated model (SLE model with online learning module).

**Figure 14:**
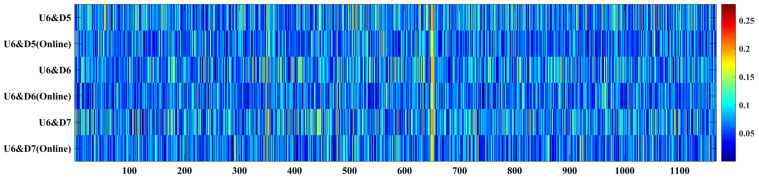
The stride-length errors of the offline model (SLE model without online learning module) and updated model (SLE model with online learning module) with different devices.

Meanwhile, the qualitative comparison of the offline SLE results and updated results on sequences #21, #22, and #23 are shown in Figure 13 and Figure 14. We find that the stride-length accuracy is significantly improved by the updated model, making the more than 80% samples’ accuracy error less than 0.1 m. The updated model results are better than the offline model, and it is robust to the different devices.

### 4.5. Walking-Distance Estimation

This section analyzes the SLE models’ cumulative walking distance estimation to evaluate the overall performance and robustness in the long-term walking scenario. The cumulative walking distance is from the number of strides and the corresponding stride-length estimation results. The walking distance is calculated as follows:

(21) $$L_{WD}=\sum_{i=1}^{N}L_{i}$$

where *N* is the number of strides and $L_{i}$ represents the SLE results of the $i^{\mathrm{th}}$ stride.

In the experiment, the dataset we applied to train models is the same as the data in Section 4.2, and the test sequences, including three additional new sequences, #21, #22, and #23, are used to analyze the performance of the walking distance estimation. Table 6 shows the error and error rate of the stride length estimation with three SLE models. It is easy to see that adaptive learning with preprocessing and online learning (marked as “Adaptive learning + PP + OL”) can significantly decrease the error and error rate, especially on the new sequences #21, #22, and #23. The reason is that the online learning module can efficiently optimize the parameter of the CNN module. Compared with direct adaptive learning and “Adaptive learning + PP”, “Adaptive learning + PP + OL” reduces the average error by nearly 8% and 4%, respectively, and decreases the error rate of walking distance by 0.3% and 0.2% on average, respectively. In terms of robustness of the model, we find that “Adaptive learning + PP + OL” is relatively stable, and the max error of walking distance from the online learning-based model is 45.59 m, which is significantly less than the other two models. The findings benefit from the outstanding online learning module of the proposed model, which mitigates the heterogeneity when the motion data from new testers or devices.

### 4.6. Comparison with Other Methods

So far, the above experiments have been conducted to demonstrate the performance of each module in the proposed model for stride-length estimation. To verify the superiority of the proposed EL-SLE model, we compare the proposed method with four established methods in terms of the error rate of stride length. The first is that proposed by Kim, who proposed an empirical model according to the average acceleration magnitude during pedestrian walking [8]; the second is from Weinberg, who proposed a conventional SLE model based on the component strategy with vertical acceleration in each pedestrian walking stride [7]; the SLE model based on recurrent neural network is proposed to solve the problem, which is used as the third compared method [50]; and the fourth compared method is based on the pedestrian mode recognition for accurate stride-length estimation during complex walking scenarios [34].

**Figure 15:**
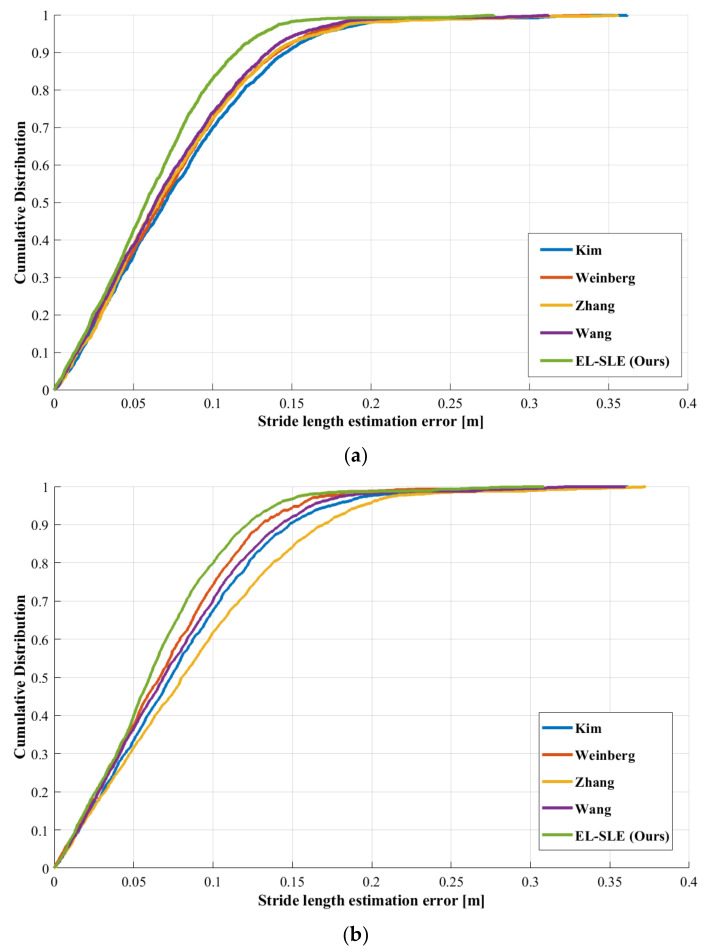
Cumulative distribution diagram of absolute errors of stride-length estimation with different SLE models on sequences #1 (**a**) and #21 (**b**).

To conduct the exhaustive experiments, we use the eight test sequences, including three unseen data, sequences #21, #22, and #23, which is challenging for learning-based methods. In the experiment, the error rate and absolute errors of stride length are used to verify the performance of SLE models. At the same time, we analyze the end points’ positioning error by using the standard PDR algorithm, which is a significant and practical index to evaluate performance. In the PDR mechanism, the models have the same heading angles, and the angles are provided by the ground truth from VIO in Section 3.3. The error rate and end points’ positioning error are shown in Table 7, and Figure 15 illustrates the cumulative distribution diagram of absolute errors of stride-length estimation. In the table, we find that the Zhang model has the worst results among these methods, and the error rate of stride length is 5.83% on #23.

Meanwhile, the end point’s positioning error of sequence #23 is more than 18 m, so the errors in stride-length estimation can lead to severe positioning deviation in the PDR mechanism. The reason is that the LSTM-based SLE model has poor generalization ability on new devices or pedestrians. On the contrary, the proposed method shows excellent performance in all sequences. The average error rate of the proposed method is 4.26%, and the proposed model obtained 8.39 m of end point’s positioning error on average. The results are thanks to the online learning module of our model, which can extract the new CNN-based features for mode recognition. Compared with Kim, Weinberg, Tapeline, and Wang, the average end points’ positioning errors were reduced by nearly 51%, 48%, 53%, and 39%, respectively. Furthermore, we find that the proposed EL-SLE model shows more stability than the other four models, and the error rate of stride-length estimation is less than 4.4%. Therefore, our model provides more accurate and robust results of stride-length estimation during complex walking modes and dynamic scenes, and the accurate results can significantly reduce positioning errors in location-based services. Figure 15 shows the cumulative distribution diagram of absolute errors of stride length estimation with different SLE models on sequences #1 and #21. The figure clearly shows that the proposed method performs better than the other methods on the selected sequences, and the performance gap between the proposed model and other models is even more evident in sequence #21. For the unseen data, the Kim, Weinberg, and Wang models can obtain more accurate results than the Zhang model, which depends on the direct LSTM. The reason is that the simple LSTM-based SLE model can only extract the temporal features in the fixed mode during walking. Nevertheless, the proposed method employed LSTM and CNN to extract the multiple motion features, providing reasonable assistance for walking mode recognition during long-term and complex movement. In addition, the parameters of CNN are optimized to process the unseen data using an online learning module. Therefore, the proposed model performs better than other models, such as the empirical, pattern recognition, and direct LSTM methods.

## 5. Conclusions

This paper proposes a learning-based stride-length estimation method to approach the challenge of device or user heterogeneity for the SLE model by using an adaptive learning module. We are the first to propose the adaptive learning strategy using LSTM and CNN to extract both temporal characteristics and statistical features from IMU data, strengthening the model’s robustness under challenging conditions. Additionally, we use denoising autoencoders and preintegration for data preparation, improving the training efficiency of neural networks. The output of two network modules is then combined with mapping the eigenvectors of the stride length. Furthermore, the online learning module is proposed to extract new features and optimize CNN parameters for the customized model. Meanwhile, we suggested a simple method that employs the built-in sensors of smartphones and requires no additional hardware, increasing the convenience of labeled data for network training. Extensive trials in challenging indoor and outdoor environments are conducted to assess the viability of the proposed EL-SLE. The experimental findings support the generalizability of the SLE model, and the results demonstrate that our approach is capable of superior stride-length estimates than the other models. Our method’s average stride-length error rate is 4.26%, significantly less than the state-of-the-art SLE methods. We analyze the application of SLE for PDR-based pedestrian positioning, and the results show that the proposed method can obtain more accurate positioning in long-term walking. Compared with other methods, EL-SLE decreases the average positioning error of endpoints by nearly 51%, 48%, 53%, and 39%, respectively. In addition to reducing the positioning error for PDR, the accurate stride-length estimation can be employed to provide better service for sports analytics, health care, and extensive data statistics for the Internet of Things.

However, some limitations need to be approached in our future work. The proposed model has been verified with experiments that involve normal walking, while the test gait does not include more special motion gait such as backing, side walking, and jumping. Moreover, the proposed model needs to be processed by a portable computer during online learning, and the process takes a little time when the data is transmitted between the mobile devices and the server, leading to delay problems during step estimation and the obstacle of practical application. In the future, the stride-length estimation model needs to be extended to test with more complex gaits and dynamic environments, and we will consider 5G and signal compression transmission technology to achieve low-delay result output in the Internet of Things.

## Figures and Tables

**Table 1:** Description of testers and devices.

Testers	**Abbreviation**	**Gender**	**Age**	**Height (cm)**
	T1	Male	26	178
	T2	Male	27	174
	T3	Female	23	162
	T4	Female	25	165
	T5	Male	29	181
Devices	**Abbreviation**	**Model**	**Version**	**Inertial sensor**
	D1	Google Pixel XL 3	9.0	BMI160
	D2	Samsung S8	8.0	LSM6DSL
	D3	Huawei Mate 30	11.5	ICM-20690
	D4	OPPO Reno 6	11.3	ICM-40607
	D5	Xiaomi 12	12.0	AK09918

**Table 2:** Details of the test sequences in the dataset.

Sequence	User (Gender)	Device	Attachments	Time (s)	Distance (m)
# 1	# 1/M	Samsung S8	Handheld	1495	1578
# 7	# 2/M	Huawei Mate 30	Handbag	1497	1573
# 11	# 3/F	OPPO Reno 6	Handbag	1450	1570
# 14	# 4/F	Xiaomi 12	Pocket	1462	1586
# 17	# 5/M	Samsung S8	Handheld	1457	1583

**Table 3:** Effect of data preprocessing.

Test Seq.	L-SLE		L-SLE + DA		L-SLE + DA + PI	
	Mean Error (m)	Error Rate	Mean Error (m)	Error Rate	Mean Error (m)	Error Rate
# 1	0.067	4.32%	0.063	4.30%	0.063	4.29%
# 7	0.065	4.30%	0.062	4.29%	0.060	4.28%
# 11	0.059	4.28%	0.057	4.25%	0.054	4.24%
# 14	0.054	4.26%	0.053	4.23%	0.052	4.22%
# 17	0.062	4.29%	0.057	4.26%	0.058	4.26%
Ave.	0.061	4.29%	0.058	4.27%	0.057	4.23%

**Table 4:** The performance of the different neural networks.

Test Seq.	LSTM		CNN		LSTM + CNN	
	MAX Error (m)	Error Rate	MAX Error (m)	Error Rate	MAX Error (m)	Error Rate
# 1	0.334	4.47%	0.428	4.76%	0.326	4.29%
# 7	0.327	4.46%	0.426	4.74%	0.320	4.28%
# 11	0.328	4.43%	0.419	4.71%	0.314	4.24%
# 14	0.327	4.47%	0.422	4.68%	0.309	4.22%
# 17	0.331	4.51%	0.431	4.75%	0.316	4.26%
Ave.	0.329	4.47%	0.425	4.73%	0.317	4.26%

**Table 5:** Accuracy comparison of the offline model (SLE model without online learning module) and updated model (SLE model with online learning module).

Test Seq.	Tester	Device (IMU)	Offline Model		Updated Model	
			Mean Error (m)	Error Rate	Mean Error (m)	Error Rate
# 21	T #6	Vivo X70 (LSM6DSO)	0.068	4.33%	0.057	4.25%
# 22	T #6	Xiaomi 10 (LSM6DS0)	0.066	4.31%	0.055	4.24%
# 23	T #6	Galaxy F52 (MMC5603)	0.072	4.37%	0.064	4.29%
Ave.			0.068	4.34%	0.059	4.26%

**Table 6:** Performance comparison of walking distance with different models, including Adaptive learning, Adaptive learning with Preprocessing (Adaptive learning + PP), and Adaptive learning with Preprocessing and Online Learning (Adaptive learning + PP + OL).

Model	Attributes	Seq#1	Seq#7	Seq#11	Seq#14	Seq#17	Seq#21	Seq#22	Seq#23	Ave.
Adaptive learning	Error (m)	45.62	42.37	39.31	37.97	42.68	50.97	51.36	51.08	45.17
	Error rate (%)	2.9	2.7	2.5	2.4	2.7	3.2	3.3	3.1	2.9
Adaptive learning + PP	Error (m)	43.75	40.55	39.15	36.51	40.09	48.28	49.96	50.77	43.63
	Error rate (%)	2.8	2.6	2.5	2.3	2.5	3.1	3.1	3.2	2.8
Adaptive learning + PP + OL	Error (m)	43.63	39.43	39.24	35.94	39.81	45.57	44.38	45.59	41.70
	Error rate (%)	2.8	2.5	2.5	2.3	2.5	2.8	2.8	2.9	2.6

**Table 7:** Performance of stride-length estimation with different SLE models.

Methods	Attributes	Seq #1	Seq #7	Seq #11	Seq #14	Seq #17	Seq #21	Seq #22	Seq #23	Ave.
Kim [8]	Error rate (%)	5.35	5.34	4.74	4.70	5.29	5.33	5.31	5.38	5.18
	Positioning error (m)	17.80	17.62	16.68	16.92	17.35	18.16	17.07	17.83	17.43
Weinberg [7]	Error rate (%)	5.03	4.96	4.77	4.70	5.16	5.09	5.17	5.11	5.00
	Positioning error (m)	16.33	15.75	14.97	14.81	16.56	16.38	16.75	17.92	16.18
Zhang [50]	Error rate (%)	5.36	5.23	5.16	5.08	5.54	5.78	5.72	5.83	5.46
	Positioning error (m)	17.82	17.14	17.09	16.97	18.42	18.89	18.75	19.01	18.01
Wang [34]	Error rate (%)	4.57	4.42	4.39	4.36	4.53	4.52	4.56	4.49	4.48
	Positioning error (m)	13.14	12.79	12.37	12.27	13.05	13.10	13.22	13.08	12.88
EL-SLE (ours)	Error rate (%)	4.31	4.28	4.25	4.21	4.26	4.25	4.24	4.29	4.26
	Positioning error (m)	8.54	8.37	7.92	7.63	8.83	8.97	7.80	9.03	8.39
